# Dynamic changes of central thyroid functions in the management of Cushing's syndrome

**DOI:** 10.20945/2359-3997000000019

**Published:** 2018-03-23

**Authors:** Sema Ciftci Dogansen, Gulsah Yenidunya Yalin, Bulent Canbaz, Seher Tanrikulu, Sema Yarman

**Affiliations:** 1 Istanbul University Istanbul University Istanbul Faculty of Medicine Department of Internal Medicine, Division of Endocrinology and Metabolism Istanbul Turkey Istanbul University, Istanbul Faculty of Medicine, Department of Internal Medicine, Division of Endocrinology and Metabolism, Istanbul, Turkey

**Keywords:** Cushing's syndrome, thyroid dysfunction, syndrome of inappropriate secretion of TSH, endogenous hypercortisolemia, central hypothyroidism

## Abstract

**Objective:**

The aim of this study was to determine the frequency of central thyroid dysfunctions in Cushing's syndrome (CS). We also aimed to evaluate the frequency of hyperthyroidism due to the syndrome of the inappropriate secretion of TSH (SITSH), which was recently defined in patients with insufficient hydrocortisone replacement after surgery.

**Materials and methods:**

We evaluated thyroid functions (TSH and free thyroxine [fT^4^]) at the time of diagnosis, during the hypothalamo-pituitary-adrenal axis recovery, and after surgery in 35 patients with CS. The patients were separated into two groups: ACTH-dependent CS (group 1, n = 20) and ACTH-independent CS (group 2, n = 15). Patients’ clinical and laboratory findings were evaluated in five visits in the outpatient clinic of the endocrinology department.

**Results:**

The frequency of baseline suppressed TSH levels and central hypothyroidism were determined to be 37% (n = 13) and 26% (n = 9), respectively. A negative correlation was found between baseline cortisol and TSH levels (r = -0.45, p = 0.006). All patients with central hypothyroidism and suppressed TSH levels showed recovery at the first visit without levothyroxine treatment. SITSH was not detected in any of the patients during the postoperative period. No correlation was found between prednisolone replacement after surgery and TSH or fT4 levels on each visit.

**Conclusion:**

Suppressed TSH levels and central hypothyroidism may be detected in CS, independent of etiology. SITSH was not detected in the early postoperative period due to our adequate prednisolone replacement doses.

## INTRODUCTION

The major regulators of TSH secretion are commonly known as the stimulation effect of TRH and the negative feedback of the fT4 and fT3. However several factors such as dopamine and somatostatin, also play a role in the modulation of TSH secretion and the secretion pattern demonstrates a diurnal rhythm via these regulators (1). The hypothalamus-pituitary-thyroid (HPT) axis may be altered in Cushing's syndrome (CS). Both endogenous CS and exogenous hypercortisolism suppress serum TSH levels (2–10). Hypercortisolemia decreases the TSH pulse amplitude and nocturnal surge without causing any changes in the TSH pulse frequency (4–6,8). Furthermore, many studies have indicated that hypercortisolemia blunts the TSH response to TRH (2,4,10–12). TSH suppression in hypercortisolemia is most likely related to decreased TRH gene expression (13). However, the presence of hypercortisolemia may also decrease TSH secretion by having a direct effect on the pituitary thyrotropin cells through annexin-1, somatostatin, leptin, and dopamine (4,14–17). Another possible mechanism in the TSH suppression of glucocorticoids is the type 2 deiodinase enzyme activity that converts T4 to T3 in the hypothalamus and pituitary. The local T3 levels in the hypothalamus and pituitary are also important in the regulation of the HPT axis. Increased type 2 deiodinase activity due to glucocorticoids causes increased local T3 levels which eventually leads to the suppression of TRH and TSH secretion (18,19). In addition, central hypothyroidism with reduced fT4 may be present in patients with hypercortisolism (6,7). Mathioudakis and cols. (7) reported that central hypothyroidism can be seen in patients with ACTH-secreting pituitary microadenomas with a prevalence as high as 18%.

Recently, the syndrome of the inappropriate secretion of TSH (SITSH) was reported as a clinical condition with the presence of normal or elevated TSH secretion despite inappropriately high levels of thyroid hormones in patients who receive insufficient hydrocortisone replacement following surgery for CS (19). Furthermore, SITSH is considered as the main cause of steroid withdrawal syndrome (SWS) (20).

In light of these reports, we aimed to determine the frequency of central thyroid dysfunctions in patients who underwent endogenous hypercortisolism. We evaluated thyroid function tests at baseline in the time of CS diagnosis and during the period of hypothalamo-pituitary-adrenal (HPA) axis recovery following surgery for CS and at remission.

## MATERIALS AND METHODS

This is a retrospective observational study from a university hospital outpatient clinic. We identified the patients (n = 35) with a confirmed diagnosis of CS who had a record of thyroid function tests during the past 10 years. All of the procedures were applied in accordance with the Declaration of Helsinki. The diagnosis of CS was based on the clinical and radiological findings (pituitary adenoma, adrenal adenoma, or bronchial carcinoid tumor confirmed by sellar or abdominal magnetic resonance imaging [MRI], lung computed tomography [CT] or ocreotide scintigraphy) and laboratory tests. The diagnosis of CS was confirmed by failure to suppress plasma cortisol levels after the administration of 1-mg-overnight and low-dose dexamethasone suppression tests (48 hours, 2 mg/day) in accordance with the current guideline (21). A definitive diagnosis of Cushing's disease (CD) was made with positive immunostaining for the ACTH of the pituitary adenoma and clinical cortisol dependency for several months after adenomectomy. The diagnosis of ectopic ACTH syndrome (EAS) was based on high plasma ACTH levels, the presence of a lung lesion on high-resolution CT scanning or ocreotide scintigraphy, histological confirmation of the tumor with positive immunostaining for ACTH, and clinical cortisol dependency during the follow-up period after tumor resection. The diagnosis of primary adrenal CS was based on the absence or diminished dexamethasone suppression of serum cortisol, a low or undetectable plasma ACTH concentration, the presence of a unilateral adrenal adenoma on CT or MRI scanning, and histological confirmation of the adenoma.

Patients with known thyroid disease at the time of CS diagnosis, patients who developed thyroid disease (including autoimmune thyroid disease) during followup, and patients who were on drugs known to alter thyroid functions were excluded. Macroadenoma (≥ 10 mm) of the pituitary, a history of conventional radiotherapy (RT) for the pituitary, and the development of hypopituitarism after pituitary surgery were also among the exclusion criteria of the study.

Among the patients who underwent pituitary surgery, adrenalectomy, or bronchial carcinoid resection, those who fulfilled the initial surgical remission criteria were included. Patients with a residual tumor after an unsuccessful initial operation or late relapse were excluded.

Initial surgical remission was defined as morning serum cortisol levels less than 2 µg/dL within a week of cortisol-secreting tumor resection. Late remission was defined as cortisol suppression with a 1-mg-overnight dexamethasone test following HPA axis recovery and the discontinuation of a glucocorticoid (prednisolone) replacement. HPA axis recovery was evaluated by using morning cortisol and/or ACTH stimulation tests. Prednisolone replacement was discontinued when morning plasma cortisol levels were ≥ 10 µg/dL or stimulated cortisol levels were approximately ≥ 18 µg/dL with ACTH stimulation test (22). Prednisolone replacement was gradually tapered and discontinued following HPA axis recovery.

We analyzed the correlation of serum cortisol with TSH and fT4 levels with in patients who had hypercortisolism due to CD (n = 17); EAS (n = 3); or primary-adrenal CS (n = 15). Comparisons were conducted before and after the remission with surgical treatment to evaluate the changes in TSH and fT4 due to the aberrations in the HPT axis.

Serum cortisol, TSH (0.27-4.2 µU/Ml, intra-assay CV = 3.8%, inter-assay CV = 1.4%) and fT4 (12-22 pmol/L, intra-assay CV = 2.1%, inter-assay CV = 1.7%) levels were measured using an electrochemiluminescent immunoassay (Roche Hitachi). Serum ACTH (0-46 pg/mL, intra-assay CV: < 10%, inter-assay CV: < 10%) levels were measured using a chemiluminescence immunoassay (Immulite 2000).

Sex, age at diagnosis, mean duration of CS, duration of follow-up, baseline cortisol, ACTH, TSH, and fT4 levels, and the baseline adenoma diameter were evaluated retrospectively for each patient. Serum cortisol levels were recorded in the early postoperative period. Patients’ clinical findings and laboratory tests were evaluated in a total of five visits; the first visit was between the first and the third month; the second visit was at the sixth month; the third visit was at the 12^th^ month; the fourth visit was at the time of HPA axis recovery; and the fifth visit was the last visit of the follow-up period. Cortisol, TSH, fT4, and the prednisolone replacement dose (for patients who were on prednisolone replacement) were recorded during each visit.

ACTH-dependent (group 1) and ACTH-independent (group 2) CS patients were compared in terms of the baseline characteristics and thyroid function tests during follow-up.

Statistical analyses were performed using SPSS version 21.0. Categorical variables were defined by frequency and percentage rate, and numeric variables by mean ± standard deviation (SD). In dual independent group comparisons, the student's t test was used for normally distributed continuous variables, and the Mann-Whitney U test for non-normally distributed data. Categorical variables were compared using the chisquare test. Correlation analyses were performed using the Pearson correlation test. Statistical significance was set at p < 0.05. Ranges of the correlation r value were accepted as 0-0.24 weak, 0.25-0.49 moderate, 0.50-0.74 strong, and 0.74-1.00 very strong.

## RESULTS

Thirty-five patients (28 female and seven male; mean age at diagnosis of 37.2 ± 11.4 years; range of 17-61 years) were included in the study. The mean followup time was 57.1 ± 27.6 months (range of, 21-114 months). The mean duration of CS was 2.7 ± 1.5 years (range of, 1-7 years). Group 1 consisted of bronchial carcinoid tumors (n = 3, 8%) and CD (n = 17, 49%) and group 2 consisted of cortisol-secreting adrenal adenomas (n = 15, 43%).

The mean cortisol, TSH, and fT4 levels at the initial assessment are shown in Table 1. The frequencies of baseline-suppressed TSH levels and central hypothyroidism were determined to be 37% (13/35) and 26% (9/35), respectively. The diagnosis of patients with central hypothyroidism were CD (n = 3), EAS (n = 2), and primary adrenal CS (n = 4). The baseline cortisol levels correlated negatively with the baseline TSH levels (r = -0.45, p = 0.006). However, no correlation was found between the baseline cortisol and fT4 levels (p = 0.183). In addition, no differences were found between the baseline cortisol levels of patients with and without central hypothyroidism (p = 0.218).

**Table 1:** The mean cortisol, TSH, fT4 levels and prednisolone replacement dose* on each visit

	Cortisol levels (µg/dL) mean ± SD (range)	TSH levels (µU/mL) mean ± SD (range)	fT4 levels (pmol/L) mean ± SD (range)	Prednisolone replacement dose* (mg/day) mean ± SD (range)
Baseline	26.3 ± 8.7 (13-46)	0.8 ± 0.7 (0.1-2.9)	13.8 ± 2.7 (9.3-19.7)	-
First visit	0.9 ± 1.1 (0.1-4.7)	2.1 ± 1.2 (0.5-4.7)	15.9 ± 2.2 (12.8-20.7)	7.3 ± 1.5 (5-10)
Second visit	2.9 ± 2.8 (0.1-8.3)	2.4 ± 1.3 (0.3-1.8)	15.1 ± 1.8 (12.4-18.6)	4.6 ± 1.5 (2.5-7.5)
Third visit	8.9 ± 5.8 (0.3-18.6)	2.7 ± 1.2 (0.5-4.8)	15.2 ± 1.3 (12.5-18.1)	3.6 ± 1.1 (2.5-5)
Fourth visit	13.2 ± 1.7 (10.4-16.9)	2.5 ± 1.2 (0.7-4.8)	15.1 ± 1.6 (12.7-19.1)	-
Fifth visit	15.3 ± 2.9 (10.7-21)	2.3 ± 1.1 (0.9-4.7),	15.6 ± 1.8 (12.1-20)	-

* Prednisolone replacement doses were revised for patients who were receiving prednisolone replacement after surgical treatment. Baseline: at initial assessment, First visit: Between the 1^st^ and the 3^rd^ months; Second visit: On the 6^th^ month; Third visit: On the 12^th^ month; Fourth visit: At the time of HPA axis recovery; Fifth visit: Last visit of the follow-up period.

**Figure 1:**
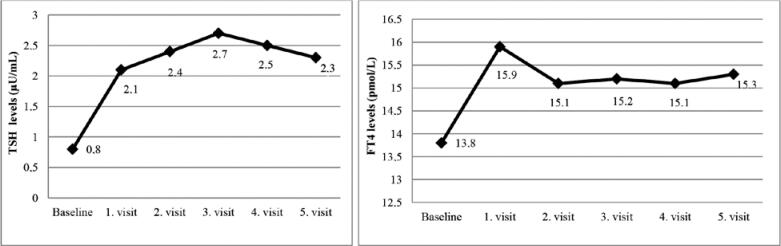
Changes in TSH and FT4 levels during folliw-up. First visit: Between the 1^st^ and the 3 ^rd^ months; Second visit: On the 6^th^ month; Third visit: On the 12^th^ month; Fourth visit: At the time of HPA axis recovery; Fifth visit: Last visit of the follow-up period.

The mean postoperative early cortisol levels of the patients were 0.7 ± 0.6 µg/dL (range of, 0.1-1.8 µg/dL). Thus, all of the patients achieved remission after surgery, and prednisolone replacement was initiated. All of the patients with central hypothyroidism were recorded as euthyroid on the first visit without the replacement of levothyroxine. The mean cortisol, TSH, and fT4 levels and prednisolone replacement dose (for patients who were on prednisolone replacement) during each visit are shown in Table 1. No correlation was found between prednisolone replacement and TSH or fT4 levels during each visit (p > 0.05). The mean HPA axis recovery time was 16.2 ± 13.2 months (range of 7-60 months). TSH levels during the last visit were significantly higher than the baseline TSH levels, and no differences in the fT4 levels were found between baseline and the last visit (p = 0.003, p = 0.295, respectively). Changes in the TSH and FT4 levels during follow-up are shown in Figure 1.

In group 1, the mean diameter of the pituitary adenoma was 5.9 ± 1.6 mm (range, of 3-9 mm), and the mean baseline ACTH level was 66.6 ± 33.8 pg/mL (range of, 16-155 pg/mL). No correlation was found between the baseline TSH and ACTH levels or the diameter of the pituitary adenoma (p = 0.268, p = 0.813, respectively). In group 2, the mean diameter of the adrenal adenomas was 32 ± 11 mm (range of, 20-54 mm).

Patients in group 1 were found to be younger than those in group 2 (p = 0.03). Although baseline cortisol levels were higher in group 1, the difference was not statistically significant (p = 0.645). The frequency of central hypothyroidism at the initial assessment, and the baseline TSH and fT4 levels were not statistically different between the two groups (p = 0.912, p = 0.242, p = 0.631, respectively). The frequency of baseline-suppressed TSH levels were higher in group 1, but the difference was not statistically significant (p = 0.069). As for the TSH and fT4 levels, the TSH levels of group 2 were higher only at the second visit (p = 0.035). No statistically significant difference was found between the two groups in terms of the HPA axis recovery time and the plasma TSH; and fT4 levels (p = 0.825, p = 0.761, p = 0.841, respectively). In addition, no difference was observed between the two groups regarding the change in plasma TSH levels (Δ TSH) at the last visit (p = 0.257). The comparisons of the two groups are shown in Table 2.

**Table 2:** Comparison of ACTH-dependent and ACTH-independent Cushing's syndrome

		Group 1 (ACTH-dependent Cushing's syndrome) (n =20)	Group 2 (ACTH-independent Cushing's syndrome) (n = 15)	p
Age at diagnosis (years)		33.8 ± 11.7 (17-61)	41.8 ± 9.6 (25-58)	**0.03**
Mean ± SD				
Sex (F/M)		14/6	14/1	0.08
Duration of follow-up (months)		63.7 ± 26.7 (25-114)	46.5 ± 23.8 (21-90)	0.06
Mean ± SD				
Baseline cortisol levels (µg/dL)		28.9 ± 9.6 (14.2-46)	22.8 ± 5.9 (13.3-32)	0.645
Mean ± SD				
Baseline TSH levels (µU/mL)		0.6 ± 0.6 (0.1-2.6)	1.2 ± 0.8 (0.2-2.9)	0.242
Mean ± SD				
Frequency of baseline suppressed TSH levels [n, (%)]		10 (50)	3 (20)	0.069
Baseline FT4 levels (pmol/L)		13.9 ± 2.8 (9.3-19.3)	13.6 ± 2.4 (10.3-19.7)	0.631
Mean ± SD				
Patients with central hypothyroidism atinitial assessment (n; %)		5 (25)	4 (27)	0.912
Early postoperative cortisol levels (µg/dL)		0.6 ± 0.4 (0.1-1.3)	0.8 ± 0.7 (0.1-1.9)	0.194
Mean ± SD				
First visit (Mean ± SD)				
	TSH levels (µU/mL)	2.1 ± 1.2 (0.5-4.7)	2.1 ± 1.3 (0.6-4.7)	0.950
	FT4 levels (pmol/L)	15.6 ± 2.2 (12.8-20.7)	16.5 ± 2.2 (13.4-19.5)	0.903
Second visit (Mean ± SD)				
	TSH levels (µU/mL)	2.2 ± 1.1 (0.3-4)	2.7 ± 1.4 (0.8-4.8)	**0.035**
	FT4 levels (pmol/L)	14.9 ± 1.6 (12.4-17.7)	15.1 ± 2.3 (12.5-18.6)	0.752
Third visit (Mean ± SD)				
	TSH levels (µU/mL)	2.6 ± 1.4 (0.5-4.5)	2.8 ± 1.2 (0.8-4.8)	0.317
	FT4 levels (pmol/L)	15.2 ± 1.1 (13.4-17.8)	15.2 ± 1.5 (12.5-18.1)	0.747
HPA axis recovery time (months)		13.6 ± 5.1 (7-25)	19.6 ± 19.3 (7-60)	0.825
Mean ± SD				
Fourth visit (Mean ± SD)				
	TSH levels (µU/mL)	2.4 ± 1.2 (0.7-4.5)	2.7 ± 1.3 (0.8-4.8)	0.761
	FT4 levels (pmol/L)	14.9 ± 1.7 (12.7-19.1)	15.3 ± 1.4 (13.5-17.1)	0.841
Fifth visit (Mean ± SD)				
	Cortisol levels (µg/dL)	15.1 ± 2.9 (10.7-20)	15.7 ± 3.1 (10.7-21)	0.472
	TSH levels (µU/mL)	1.9 ± 1.1 (0.5-4.5)	2.8 ± 1.2 (0.5-4.7)	0.458
	FT4 levels (pmol/L)	16.1 ± 1.8 (13.2-20)	14.9 ± 1.5 (12.1-17.1)	0.554
Δ TSH levels° (µU/mL)		1.3 ± 1.1 (0.2-3.4)	1.7 ± 0.9 (-0.1-3)	0.257
Mean ± SD				

Bold values are statistically significant (p < 0.05), °Δ TSH: The change in TSH levels between baseline and the last visits. First visit: Between the 1^st^ and the 3^rd^ months; Second visit: On the 6^th^ month; Third visit: On the 12^th^; Fourth visit: At the time of HPA axis recovery; Fifth visit: Last visit of the follow-up period.

## DISCUSSION

In this study, we demonstrated the effect of hypercortisolism on thyroid functions in patients with CS. Because it is common knowledge that plasma T3 levels decrease through peripheral type 1 deiodinase enzyme inhibition, we aimed to show the central effects of hypercortisolism on thyroid functions (23). Furthermore, glucocorticoids are used in the treatment of hyperthyroidism due to these peripheral effects (24). We have shown suppressed baseline TSH levels in patients with active CS, which is also compatible with a number of studies in the literature (2,3,5–7,10). In this study, a very selective population was evaluated to detect merely the effect of hypercortisolism on thyroid functions, contrary to the studies in the literature. Patients who had secondary conditions that could affect thyroid function tests, such as autoimmune thyroid disease, the presence of pituitary macroadenoma or RT were not included in the study population. Although the most important factor affecting TSH secretion in CS is considered to be hypercortisolism itself, various other factors associated with CS have been investigated as well, such as severe additional disease, goiter, and diabetes mellitus (10). Similarly in this study we observed an inverse relationship between cortisol levels and TSH levels which was compatible with many other studies (6,10–12). Furthermore, it is known that glucorticoid administration in healthy individuals also may cause dose-dependent TSH suppression (9). However, one study in the literature indicated that serum cortisol levels and TSH and fT4 levels did not show a significant correlation. That study indicated that TSH suppression in CS might be related to high cumulative cortisol levels and it is rather difficult to detect such an effect. The authors suggested that high ACTH levels in CD patients might have an effect on thyroid function tests independent of plasma cortisol levels. However, such a relationship was not proved in their study (7). Therefore, we also included an evaluation of the association between ACTH and TSH levels in our patients with ACTH-dependent CS, which has not been previously mentioned in the literature. However, we did not find any significant correlation between ACTH and TSH levels. A comparison between the isolated effects of local pituitary ACTH secretion and the ectopic secretion of ACTH would be possible only with the selective classification of two groups with either EAS or CD. However, due to the low number of EAS patients, we were unable to design such a comparison in our study. Even though our study population included only microadenoma lesions, we also evaluated the mass effect of pituitary adenoma on TSH levels and found no significant correlation between adenoma size and TSH levels.

Lower plasma T4 levels due to supressed TSH levels may be expected in hypercortisolism. However, in our study we observed central hypothyroidism in only 26% of patients and normal plasma fT4 levels in most of the patients during hypercortisolism. Mathioudakis and cols. (7) reported an even lower prevalance of central hypothyroidism (18%). Relatively normal plasma fT4 levels in hypercortisolism may be explained with the increased biological activity of TSH through posttranslational processes (25). Although the baseline fT4 levels were lower than fT4 levels during the last visit; no statistically significant difference was found. Furthermore, the cortisol levels were similar in patients with or without central hypothyroidism, and also, no correlation was found between the baseline cortisol and fT4 levels. Thus, the presence of hypercortisolemia essentially affects plasma TSH levels, and as fluctuations in fT4 levels are independent of serum cortisol, predicting which patients will develop low fT4 levels or central hypothyoridism is difficult. The factors effective in this process may be related to the sensitivity of thyrotrop cells for TSH or the iodine status of the body. Even though the iodinization of household salt has been mandatory since 1999, a recent study demonstrated urinary iodine defficiency in the 23% of the adult population in our country (26). This may be the reason for the higher prevelance of central hypothyroidism in our study compared with Mathioudakis and cols. (7). Nevertheless, even patients with central hypothyroidism generally do not have clinically evident hypothyroidism. As the clinical presentation is silent and thyroid dysfunction is assumed to recover with the improvement of hypercortisolism, levothyroxine replacement in the preoperative period is not recommended (7,22). In the literature, only a few patients received levothyroxine replacement before surgery, all of whom presented with hypothyroidism as the first finding of CS (27,28). In our series levothyroxine replacement was not given to any of the patients.

Altough the effect of CS on thyroid function tests is widely studied, studies involving the long time clinical course of these patients after surgery are limited (6,7,10,19,20,29). It has been demonstrated that TSH and thyroid hormone levels start to recover in the first six months after surgery, and these changes may take place as early as two weeks, especially in the first month following surgery (7,10,20,30). In addition, in our series we observed the most significant increase in TSH levels in the first visit, with the levels gradually increasing until the 12^th^ month of follow-up. Likewise, Roelfsema and cols. (6) assessed TSH and thyroid functions for a mean of 6.8 years after surgery for CD and found increased basal TSH secretion compared with the control group.

To evaluate tyroid function changes according to the etiologies, we divided our patients into two groups: ACTH-dependent and ACTH-independent. No significant difference was found between the hormone levels at baseline and the clinical findings at the initial assessment. However the patients with ACTH-dependent CS were younger. The presence of central hpothyroidism and low fT4 levels are more frequently reported in CD (6,7). However, we found a similar frequency of central hypothyroidism in both groups. At the follow-up visits, the mean TSH levels were slightly higher in group 2 in the second visit, which we interpreted as an incidental finding. The results of our study showed that changes in thyroid function tests in CS are the result of hypercortisolism itself independent of the etiology of CS. No significant difference was found between the two groups in terms of cortisol levels during the baseline and follow-up visits. Bartalena and cols. (12) demonstrated similar results in thyroid function tests between ACTH-dependent or ACTH-independent groups.

Even though CS and TSH supression have been well known for a long time, hyperthyroidism due to TSH secretion after surgery for CS was recently defined in the literature. This situation is described as a novel cause of SITSH (19). A TSH-secreting pituitary adenoma and resistance to the thyroid hormone are the primary causes of SITSH (31). First, Tamada and cols. (19) reported two patients who presented with SITSH caused by a decrease in the hydrocortisone replacement dose after surgery for CS. Especially the rapid decreases in the hydrocortisone replacement doses in the early postoperative period were held responsible for this process. The study also discussed that sypmtoms of hyperthyroidism due to SITSH might have overlapped with symptoms of SWS. Because of this knowledge in literature, we evaluated the prednisolone replacement doses of all patients during each visit and did not detect the presence of SITSH. This may be explained with the higher glucocorticoid replacement doses in our study compared with the series in Tamada and cols. (19). In that study, hyperthyroidism was especially detected in the first month of follow-up when the daily hydrocortisone replacement doses were ≤ 20 mg. In our study patients received a mean of 7.3 mg/day of prednisolone replacement during the first visit. In light of these findings we suspect that a relationship might exist between glucocorticoid replacement doses and plasma TSH and fT4 level, a subject on which we could not find any comments in the current literature. However we could not demonstrate a significant correlation between the prednisolone replacement and the concurrent plasma TSH and fT4 levels until HPA axis recovery in the postoperative period. Our comment regarding this is that the mechanisms leading to SITSH may not be triggered as long as the glucocorticoid replacement doses are sufficient. Tamada and cols. also reported that hypocortisolemia was responsible for the onset of the syndrome (19). Because hypocortisolemia leads to decreased type 2 deiodinase activity resulting with declining in the local hypotalamo-pituitary T3 levels, the TSH levels are elevated independent of the peripheral thyroid hormone status (18–20). However this theory is not the only explanation because presence of hyperthyroidism would then be expected in all hypocortisolemic patients. Although baseline TSH levels are elevated in patients with adrenal defficiency, hyperthyroidism is not defined in these patients (32). Our explanation is that in patients with CS plasma deiodinase activity which is sensitive to local T3 levels develops new set-points in the active hypercortisolemic period of CS, and a certain amount of time is needed for the recovery of the initial set points. Glucocorticoid replacement should be decreased gradually until the initial set points are recovered as Tamada and cols. (20) demonstrated in another prospective study that hyperthyroidism due to SITSH was also triggered by SWS after the treatment of CS. Thus, gradual reductions in steroid replacement doses are important in the prevention of both SWS and hyperthyroidism due to SITSH.

In conclusion, plasma TSH or T4 levels may be affected in CS independent of etiology. The primary cause of this finding is hypercortisolism itself. However, the particular mechanism of these processes is not clear and needs to be further evaluated. Furthermore, central hypothyroidism may be detected in these patients. Treatment recommendations need to be evaluated individually for each patient. In contrast to the general belief that TSH levels are initially suppressed in CS, which eventually recover after CS treatment and that the follow-up of thyroid function tests is not mandatory, we suggest that patients should be monitored in the postoperative period especially concerning hyperthyroidism due to SITSH. To prevent such an effect, we suggest avoiding rapid decreases in glucocorticoid replacement doses especially in the early postoperative period. The recovery of the HPT axis which was affected from glucocorticoid excess, may require some time, just as a certain period of time is necessary for the recovery HPA axis.
